# Attentional and emotional brain response to message framing in context of green marketing

**DOI:** 10.1016/j.heliyon.2020.e04912

**Published:** 2020-09-23

**Authors:** Muhammad Zubair, Xiaoyi Wang, Sidra Iqbal, Muhammad Awais, Ruining Wang

**Affiliations:** a Department of Marketing, School of Management, Zhejiang University, Room No 1206, Dormitory-D, Zijingang Campus, Zhejiang University, Hangzhou, China; b Department of Marketing, School of Management, Zhejiang University, 3^rd^ Floor, School of Management Building, Zijingang Campus, Zhejiang University, Hangzhou, China; c Department of Psychology, Shaheed Benazir Bhutto Women University Peshawar, Pakistan; d Department of Data Science and Engineering Management, School of Management, Zhejiang University, Room No 1023, Dormitory-D, Zijingang Campus, Zhejiang University, Hangzhou, China; e Department of Marketing, School of Management, Zhejiang University, Room No 508, Dormitory-A, Zijingang Campus, Zhejiang University, Hangzhou, China

**Keywords:** Attention, Emotion, Event-related brain potentials, Green marketing, Message framing, ERPs, Consumer attitude, Research and development, Consumer psychology, Decision analysis, Cognition

## Abstract

**Background:**

Message framing plays an important role in advertising strategies and has been studied from various perspectives in different behavioral studies.

**New method:**

This study employs the event-related potential technique to examine attentional and emotional brain processing as influenced by message framing in the context of green marketing.

**Results:**

The behavioral results demonstrated that purchase preference was higher under positive framing compared to negative and neutral framing.

As per the event-related potential results, negative framing elicited a larger P1 component, which reveals that in the first stage of processing information, threatening information attracted more attention. In the second and third stage, N170 and P3, respectively, were higher for positive framing, demonstrating that there was more attention toward the processing of non-threatening emotional information.

Comparison with existing method: Message Framing has been previously examined with behavioral methods. We for the first time examined it with a neuroscientific method like Event Related Brain Potential technique in a green marketing context.

**Conclusion:**

Our results compared to behavioral studies provide stronger evidence from underlying neural perspective for how message framing can be affected by attentional and emotional brain responses in the context of green marketing.

## 1 Introduction

In modern-day business, many companies pay close attention to environmental concerns, acknowledge the significance of environmentally focused marketing strategies, and emphasize “green” advertising (i.e., advertising that targets environmentally conscious consumers) for their products and services (Green Marketing: A Global Strategic Business Report - Asia Green Buildings, 2019). Green advertising has received promising appraisals (Chan et al., 2006), and has been shown to strongly affect consumers' emotional processing and to stimulate “green purchase intentions” (Carrus et al., 2008; Chang and Lee, 2009; Maheswaran and Meyers-Levy, 1990).

Among the various advertising strategies in existence, message framing is an approach that is often used to influence consumers' behaviors and attitudes (Maheswaran and Meyers-Levy, 1990) and, according to prospect theory, to objectively present information in terms of either the advantages gained or disadvantages encountered (Chang and Lee, 2009; Gerend and Cullen, 2008; Krishnamurthy et al., 2001; Levin et al., 1998; Tversky and Kahneman, 1981). Message framing is common in advertising, with positive and negative message framing now representing leading advertising strategies (Rothman et al., 2006; Tsai, 2007). For green advertising, companies, when seeking to create a positively framed message, emphasize the environmental benefits associated with purchasing the products in question; meanwhile, when creating a negatively framed message, companies highlight potential destructive outcomes that may result from the purchase of non-green products (Chandy et al., 2001; Homer and Yoon, 1992; Levin et al., 1998). Many previous studies regarding green-product-related communication have reported that negative message framing has greater effectiveness than does positive message framing (Olsen et al., 2014), and other researchers have reported that, for scenarios when consumers consider the negative information to be more trustworthy and significant, negative framing may be more influential than positive framing (Banks et al., 1995; Davis, 1995). Nevertheless, some researchers have suggested that positive framing has a greater impact than negative framing in terms of motivating buying decisions for transformational products like sneakers (Chang, 2008).

Positive and negative framing biases are associated with attention mechanisms (Kreiner and Gamliel, 2018). Yechiam and Hochman (2013, 2014) reported the results of risky choice framing biasness towards loss attention, and other studies have also found that gain/loss message framing modifies individuals' attention (Meyers-Levy and Maheswaran, 2004). Historically, framing effects have been clearly observed in relation to cognitive factors such as emotional response (Scheufele, n.d.). For instance, Nabi et al., (2019) found that gain/loss message framing can elicit positive and negative emotions. Emotions can facilitate association between the advertising message frames and attitudinal or behavioral effects (Bilandzic et al., 2017). In the context of climate change, gain frames have been found to provide a relatively strong emotional response; especially hope (Bresnahan et al., 2013; Yu and Shen, 2013). Meanwhile, through studying the significance of fruit and vegetable consumption Gerend and Maner (2011) found that gain- and loss-framed messages are effective for fostering both angered and fearful states.

In recent years, research of framing effects through examination of emotions has received enhanced theoretical (Nabi, 2003; Updegraff and Rothman, 2013) and empirical attention (Lerner and Keltner, 2001; Marti et al., 2018). Examinations of framing effects with respect to attention and emotions generally focus on cognitive bias, but certain previous studies have examined such framing effects from a range of behavioral perspectives. Some study findings support positive and negative framing effects in relation to attention, while others support emotional aspects. On the other hand, various research studies have applied cognitive neuroscience methods to examine framing effects (Lin and Yang, 2014; Murch and Krawczyk, 2013; Yang, 2015). For instance, by using an eye-tracking method Lin and Yang (2014) found that negative frames induce a relatively larger number of active eye movements (fixation points). Meanwhile, another study that used an eye-tracking method found no moderating effect for purchase intention (Yang, 2015). Use of functional magnetic resonance imaging (fMRI) has revealed an association between positive frames and reflexive brain regions, and between negative frames and reflective brain regions (Murch and Krawczyk, 2013). Further, by applying the event-related potential (ERP) method, studies have found that positive and negative messages are processed differently and provide dissimilar purchase intentions (Jin et al., 2017).

In the present study, ERP technique is used to investigate the effect of message framing on cognitive processing and decision-making in the context of green marketing. Choosing green marketing as a context for this study is due to, message framing effect in the context of green marketing is limited to the developed world, there are less number of studies in emerging economies (Camero Montgomery, 2009; Frame and Newton, 2007). In comparison with other neuroscientific methods, ERP technique provides high temporal resolution, and is an excellent measure for tracking modulations of neural activity (Ma et al., 2012). It would be valuable, in a sense, to differentiate early perceptual reactivity from more difficult and elaborate emotional processes (Gardener et al., 2013). ERP components that occur up to 300 ms after the stimulus are associated with a stage of attention, and possibly reflect early sensual encoding of stimuli that are emotionally significant (Schupp et al., 2003, 2007). In fact, this type of effect may indicate relatively refined processing of emotional stimuli, along with a rich emotional effect, and can be viewed in higher ERP components such as the P3 and late positive potential (LPP) (Cuthbert et al., 2000). In this study, we used positive-, negative-, and neutral-framed messages as experimental stimuli. According to Jin et al. (2017) consumers' cognitive processes differ depending on the type of advertising messages they view.

Several research reports have stated that the P1 and N170 are influenced by attention processing (Batty and Taylor, 2003; Miyoshi et al., 2004). Meanwhile, other studies have reported that the P300 is related to the cognitive process of stimulus evaluation and selection (Campanella et al., 2002; Miltner et al., 2005), and that LPP is associated with evaluative categorization (Chen et al., 2010; Wang et al., 2016). Therefore, based on visual observation and various decision neuroscience studies regarding attention and emotional processing (Picton et al., 2000), in the present study we analyze data for three ERP components, the P1 (100–180 ms), N170 (130–200 ms), and P3 (300–450 ms) to determine the associated attentional and emotional mechanisms.

The P1 is the first positive-going component; it normally starts at 70–90 ms after stimulus presentation, and reaches peak level at approximately 80–130 ms, with a higher magnitude at the lateral occipital scalp areas (MANGUN, 1995). According to (Itier and Taylor, 2004), the P1 component reflects greater sensitivity and higher amplitude to faces than to other kinds of stimuli. In addition, the P1 component can be stimulated by attention to the stimulus (MANGUN, 1995).

The N170 component is related to visually evoked ERP components, and it is stimulated at approximately 170 ms after stimulus onset (Eimer, 2000). The N170 is ranked as a face-sensitive and right-lateralized ERP component because it reflects a larger amplitude for faces than non-face objects (Bötzel et al., 1995). The obvious nature of the N170 as a face-sensitive component is due to unrestrained differences in low-level visual stimulus features (Thierry et al., 2007). Some researchers have claimed that the N170 is not a face-sensitive component, but is specific to expert processing and can be stimulated by non-face stimuli (Gauthier and Curby, 2005; Rossion et al., 2002; Thierry et al., 2007). However, there are some contradicting findings regarding the N170 response to emotional expressions: some studies have reported that the N170 has no response to emotional expressions (Herrmann et al., 2002; Münte et al., 1998; Tanaka and Curran, 2001), while other studies have found that the N170 modulates emotional expression (Batty and Taylor, 2003; Eimer et al., 2003; Miyoshi et al., 2004).

The P3 component is described as a positive-going wave with a latency range of 250–450 ms at the frontal and centroparietal scalp areas; it can be modulated in task-relevant activities (Cuthbert et al., 2000). The P3 component can also be stimulated by emotional stimuli with higher amplitudes (Caharel et al., 2005; Cuthbert et al., 2000). The emotional effect on the P3 component may indicate attention towards motivationally relevant stimuli (Palomba et al., 1997).

A variety of research studies have examined the roles of attention and emotion in framing effects from different perspectives (Lin and Yang, 2014; Murch and Krawczyk, 2013), but there is, as yet, no clear synthesis of the current evidence regarding this issue. Choosing the neuroscientific method will help us to identify underlying neural mechanism, particularly how message framing influence information processing in different brain regions (Jin et al., 2017). Framing effects have been studied via neuroscientific methods by different researchers (Lin and Yang, 2014; Murch and Krawczyk, 2013; Jin et al., 2017). Application of an ERP approach can, thus, help to provide neural-potential evidence that can contribute to the construction of persuasive message designs. In fact, by using a neuroscientific method such as ERP, this paper is the first to examine the respective responses to message framing in the context of green marketing. Explicitly, our main focus is to examine the neural mechanism of message framing. Our research questions are: 1) When the potential benefits and losses of a product are portrayed through message framing, do they influence attention and emotion? and 2) Does the fabrication of such positive and negative message-framing shape purchase intentions regarding environmentally safe products? Based on the abovementioned literature, we have developed the hypothesis that the ERP components discussed above explain the effect of different message-framing types on participants' attention to and processing of emotional stimuli regarding eco-friendly products.

## 2 Method

### 2.1 Participants

In this study, we recruited 20 Pakistani male postgraduate students. The members of this sample ranged in age from 25 to 35 years (M_age_ = 29.45 years, standard deviation [SD] = 3.02 years). The participants did not have any neurological or psychiatric diseases, and had normal or corrected-to-normal vision. Before conducting the experiment, written informed consent was obtained from all participants. As a result of abnormal electroencephalogram (EEG) recordings, data for one participant were discarded. Thus, we used data for 19 participants, aged 25–35 years (M _age_ = 29.68 years, SD = 2.91 years), for this study. The protocol for this study was approved by the Ethics Committee of Neuromanagement Laboratory in the School of Management, Zhejiang University China.

### 2.2 Stimuli

We included 20 pictures of green products and three message types, positive, negative and neutral, in the experiment. All the pictures were taken from Google Internet and they were neutral. Only the message content was shaped in three forms like positive, negative and neutral. All pictures and messages were appeared randomly on computer screen. The three types of messages developed were consistent with the three-stage model of facial-expression processing (Luo et al., 2010), and the three-stage neural processing of emotional words (Zhang et al., 2013). Different research studies have applied same framing manipulation in designing their messages (Cheng et al., 2014; Hartmann et al., 2008; Janiszewski et al., 2003; Wu and Cheng, 2011). Each picture and message were presented on an independent slide. The pictures used were of daily-life green products (i.e disposable cups, shopping bags, batteries), all of which were taken from the Internet. The positive message developed was “buying this protects the environment”; the negative message was “ignoring this destroys the environment”; and the neutral message was “this is an environmentally safe product.” All of the pictures and messages were developed by the authors and were evaluated and approved by five members of the Neuromanagement Lab. To ensure a smooth flow and consistency in terms of the experiment background, a uniform size of 840 × 640 pixels was used for the product pictures, and a size of 1,300 × 420 pixels was used for the messages. We performed these sizing-related adjustments using Photoshop software.

### 2.3 Procedure

**Figure 1:**
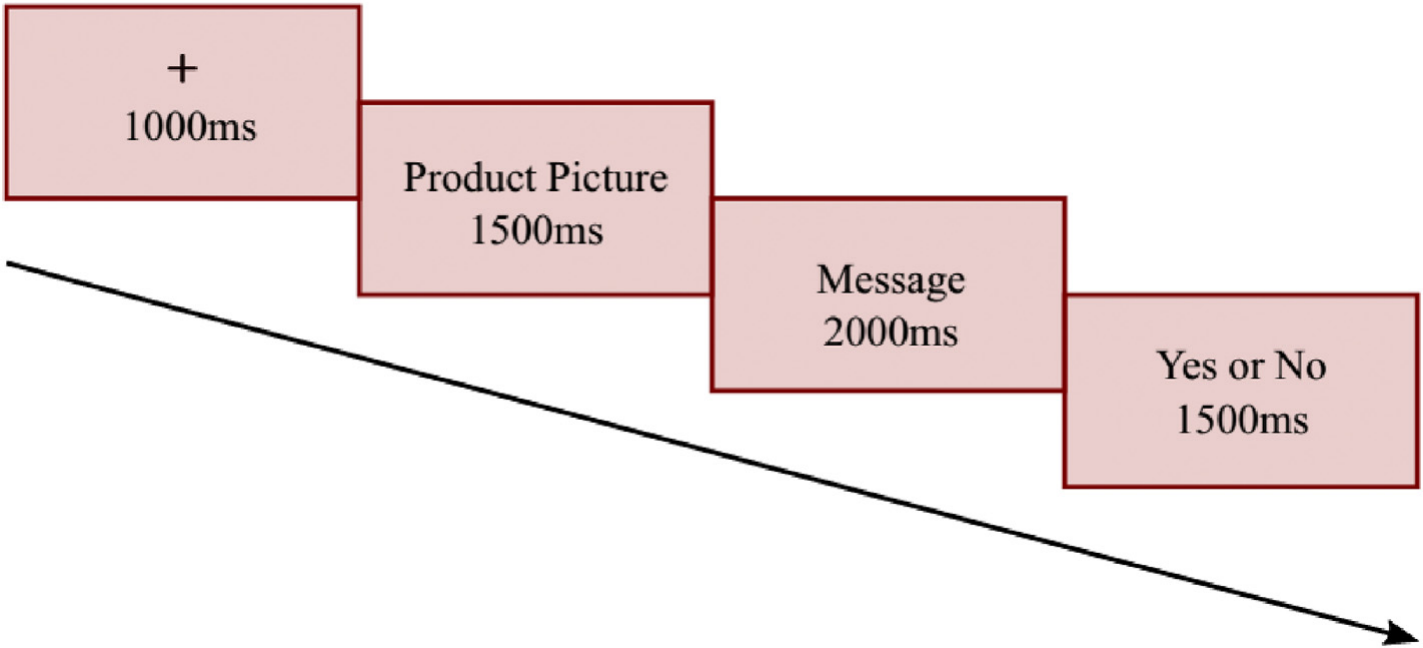
A single trial of the experiment procedure. Participants saw a green product picture first, then a message, and then recorded their choice in the end.

All of the participants received written instructions for performing the experiment. The experiment was conducted in a dimly lit, electrically shielded room. The distance between each subject and the computer screen was set at 100 cm. Participants used a keyboard to record their choices. The experiment was designed in three, unrepeated blocks. We performed a total of 140 trials in the formal experiment, and three practice trials were conducted before the experiment to allow the participants to familiarize themselves with the experimental procedure. Each experiment was conducted over approximately 15 min. The experiment was run using the E-prime 2.0 software package (Psychology Software tools, Pittsburgh, PA, USA). As shown in Figure 1, in each trial the participants viewed, in sequence, a fixation points for 1000 ms, a product picture for 1500 ms, a message for 2000 ms and, finally, a question regarding their selection, which appeared for 1500 ms. Based on the information provided in the message, the participants were asked: “Are you willing to buy the product?” The participants responded “yes” or “no”; key “1” was used for “yes,” and key “3” was used for “no.”

### 2.4 Design

This study used a 3 (message frame: positive versus negative versus neutral) × 6 (electrode sites) design, and a within-subjects factorial analysis of variance (ANOVA) to obtain statistical results for the ERP (Flaisch et al., 2008; Zhang et al., 2013). Mauchly's Test of Sphericity was used for multivariate normal distribution and Greenhouse-Geisser correction was applied when appropriate (Gardener et al., 2013). We considered p-values of less than 5% to indicate significance (Keeser et al., 2011). We implemented Bonferroni correction to adjust for multiple comparisons.

### 2.5 EEG recordings

We recorded EEG data (band-pass filter: .05–70 Hz, sampling rate: 500 Hz) using a NeuroScan SynAmps2 Amplifier (Scan 4.3.1, Neurosoft Labs, Inc., Virginia, USA). Data recording was performed on 64 scalp sites using Ag/AgCl electrodes, following the standard international 10–20 system. We recorded the electro-oculograms using electrodes located near the outer canthus of each eye (horizontal) and above and below the left eye (vertical), respectively. During the experiment, electrode impedance remained below 5 kΩ. The left mastoid was used as a reference electrode. Offline EEG data were referenced to the average of the left and right mastoids. Digital filtering of electrooculography artifacts was conducted through a zero-phase shift (low pass at 30 Hz, 24 dB/octave). The EEG level for epochs was set at between −200 ms and 800 ms. Baseline correction was performed based on the 200-ms pre-stimulus interval. Artifacts beyond ± 80 μV were rejected.

The mean amplitudes of the ERP components P1 (100–180 ms), N170 (130–200 ms) and P3 (300–450 ms) were measured at the PO5/POZ/PO6/O1/OZ/O2, P5/P3/P1/P2/P4/P6, and F3/FZ/F4/FC3/FCZ/FC4 electrode sites, respectively.

## 3 Results

### 3.1 Behavioral results

**Figure 2:**
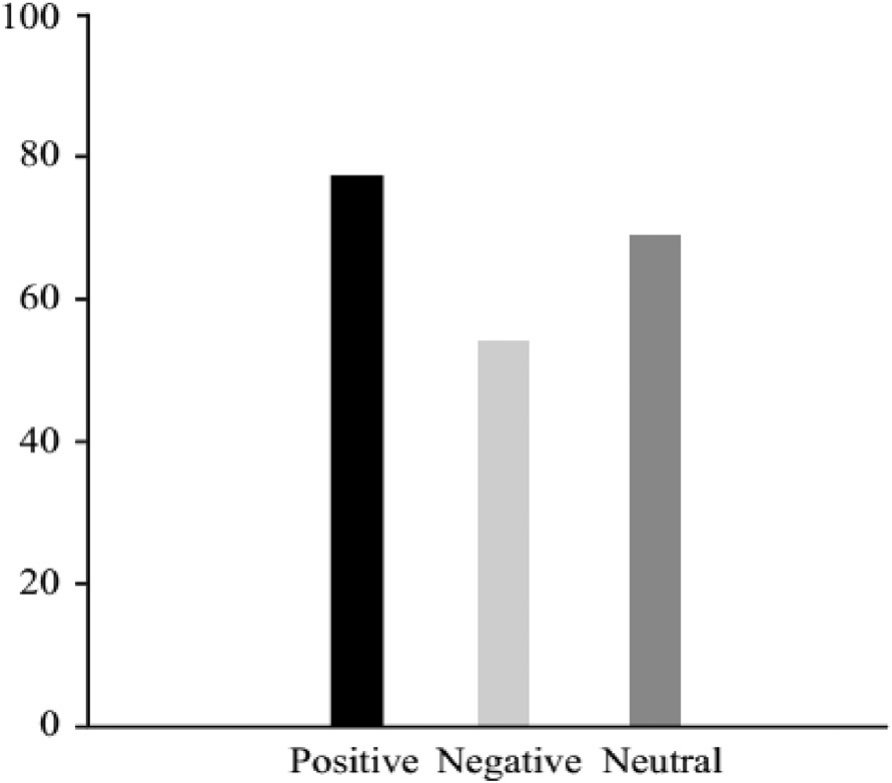
Behavioral results of the purchase intentions of participants in percentage form for positive, negative, and neutral message framing.

To compare the mean values of the three framing variables one-way ANOVA was used. According to the results of this test we found a significant effect for framing (F (2, 36) = 4.834, p < .05). The participants demonstrated higher purchase intentions for positive-framing. Figure 2 shows the participants' willingness to buy. The mean scores of willingness to buy for positive framing was (M_Positive_ = 76.98, SE = 5.711), negative framing was (M_Negative_ = 54.02, SE = 6.881) and neutral framing was (M_Neutral_ = 68.83, SE = 6.478).

### 3.2 ERP results

**Figure 3:**
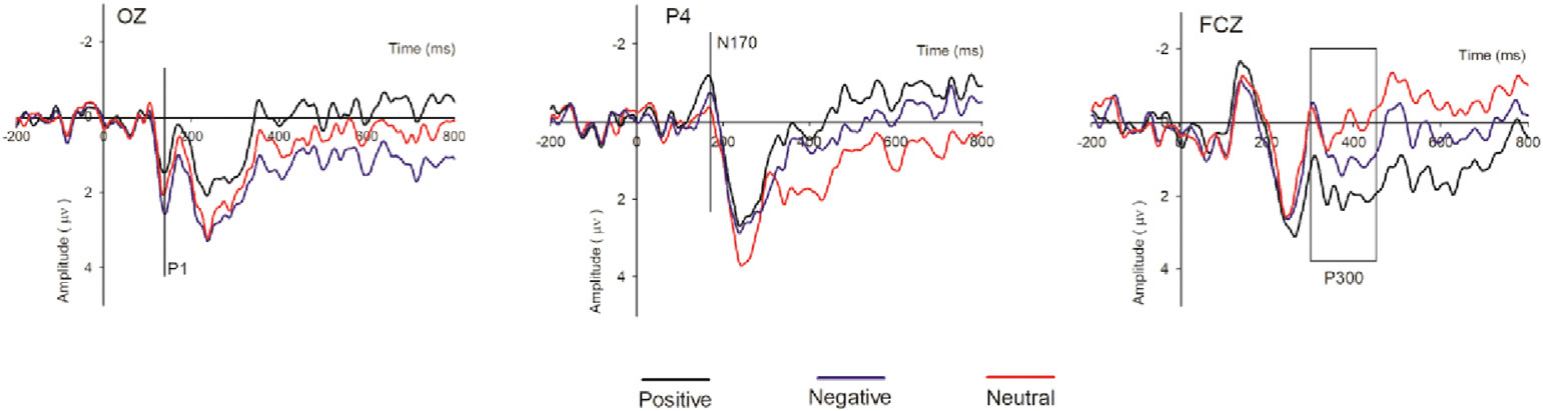
Grand average ERPs of participants for the positive message (black lines), negative message (blue lines), and neutral message (red lines) recorded at the indicated electrode sites.

*P1*. For the P1, statistical analysis of the repeated-measures ANOVA showed a significant main effect for framing effect (F (2, 36) = 4.903, p < .05) and electrodes (F (5, 90) = 7.803, p < .05). However, the interaction effect of the framing and electrodes was insignificant (F (10, 90) = .980, p > .05). Multiple comparisons produced amplitude estimates for positive framing (M_Positive_ = −.683, standard error [SE] = .457), negative framing (M_Negative_ = .541, SE = .409), and neutral framing (M_Neutral_ = .513, SE = .415), respectively (Figure 3).

*N170.* For the N170, the main effects for framing (F (2, 36) = 4.185, p < .05) and electrodes (F (5, 90) = 4.764, p < .05) were significant. However, the interaction effect of framing and electrodes (F (10,180) = 1.637, p > .05) was insignificant. Multiple comparisons produced amplitude estimates for positive framing (M_Positive_ = −2.108, SE = .393), negative framing (M_Negative_ = −.636, SE = .345), and neutral framing (M_Neutral_ = −.950, SE = .427), respectively (Figure 3).

*P3.* For the P3, the main effects for framing (F (2, 36) = 4.136, p < .05), electrodes (F (5, 90) = 19.314, p < .05) and interaction of framing and electrodes (F (10,180) = 4.351, p < .05) were all significant. Multiple comparisons showed a higher mean amplitude for positive framing (M_Positive_ = 1.998, SE = .553) than negative (M_Negative_ = .890, SE = .634) and neutral framing (M_Neutral_ = .242, SE = .563), respectively (Figure 3).

## 4 Discussion

In Study 1, we examined how attentional and emotional stimuli (see Figure 3) can evoke ERP components. Consistent with our hypotheses, the results showed that positive message framing has a significantly greater impact on the P3 and N170 components, while negative message framing has a stronger influence on the P1 component.

### 4.1 General discussion

In the current study, we applied three types of message frames, positive, negative, and neutral, in the context of green marketing to investigate attentional and emotional brain processing of marketing stimuli. For each message-frame type, the participants' willingness to buy was tested. The behavioral results indicated that the participants had higher willingness to buy when positively framed messages were used than when negatively and neutrally framed messages were used. We observed important contributions to ERP components (the P1, N170 and P3). The P1 component was highly elicited by negatively framed messages, while the N170 and P3 components were highly elicited by positively framed messages. Our findings, from a neural perspective, indicate the effect marketing-related stimuli have at different brain levels.

The subjects showed very early significant emotional effects for the P1 component at the first stage. The P1 component is commonly known to reflect early attention allocation (Zhang et al., 2013). The results of this study indicate that the amplitude for the negatively framed messages was higher than that for the positively and neutrally framed messages at parieto-occipital areas. This suggests that negative framing attracts more attention resources than do positive and neutral framing, respectively. Previous studies have reported that negative wording, which can easily attract attention or negativity bias, produces noticeable response at the P1 (Cacioppo et al., 1999; Crawford and Cacioppo, 2002). Our results support this finding, as we observed quick responses to negative stimuli, meaning such stimuli easily attract attention. In previous studies, negatively valanced stimuli have been found to induce a higher peak when compared to positive and neutral stimuli, which can result in negativity bias (Smith et al., 2003); similar results were found in this study. The improvement in the P1 component towards negative message framing may be credited to emotional processing of negative stimuli through the subcortical and cortical areas (Morris et al., 1999; Pessoa and Adolphs, 2010). In the current study, the P1 component was clearly evoked in the cortical region. Elaborating negative framing in the very early stage can help to induce participants to pay more attention to this possibly threatening stimulus. For instance, in green marketing, negative framing can cause consumers, from the outset, to pay more attention to the emotionally threatening information in the advertisement.

Analysis of the N170 component indicated that subjects showed a higher amplitude for positive framing than for negative and neutral framing. Previous studies have found larger N170 amplitude for faces than non-face objects (Rossion et al., 2000), while some other studies have found a large N170 amplitude for the visual presentation of words (Frühholz et al., 2011; Rossion et al., 2003; Simon et al., 2007). Luo et al. (2010), found a larger amplitude for facial expressions in the right hemisphere, and it is interesting that our study also found a larger N170 amplitude in the right hemisphere for message framing. Additionally, the results of a study conducted by (Caharel et al., 2005) revealed that happiness-related and non-threatening stimuli elicit the N170 component. The observed improvement in N170 towards positive message framing may be credited to emotional processing of positive stimuli. In this study, we found that the N170 component differentiates emotional stimuli when compared to neutral stimuli. The N170 component was affected by the emotional content of sentences, which are common used to create message framing in marketing. Specifically, in the context of green marketing, participants processed the information and showed a preference for potential gain by choosing the positively framed messages.

In the third stage, differentiation of the positive and negative emotional stimuli from the neutral stimuli (Luo et al., 2010) was also found. According to Fischler and Bradley (2006), a frontal-central, positive-going shift is boosted at 300–600 ms post-stimulus for pleasant and unpleasant stimuli when compared with neutral stimuli. Furthermore, we observed the largest P3 amplitude at frontal electrodes for positively framed messages (Potter et al., 2000), which is similar to previous findings (Schapkin et al., 2000; Kissler et al., 2009). Our findings indicate that the brain differentiates all three types of messages, with a higher amplitude for positive framing than for negative and neutral message framing, respectively. Our study consistently found, through analysis of P3 amplitude, processing of the emotional content of the message frames at the third stage. At this later stage, the subjects would have developed an understanding of the contents of the messages. Positive and negative message framing has emotional valence, and their contents have some conceptual meaning. The contents of the messages can induce purchase behavior by reflecting potential gains and losses in the given context of green marketing. In contrast, neutral messages have no valence and do not contain slogans that encourage among consumers deep thought regarding the message.

The present analysis found a significant effect for the various message-framing types. The messages were processed differentially and reflected non-similar purchase intentions. Although several studies have produced strong evidence for the framing effect, we focused on examining, from an underlying neural perspective, the immediate brain response in the context of green marketing. Existing behavioral studies have not identified the mechanism by which consumers perceive green marketing as good, and how positive and negative framing are effective in this regard. For this reason, we used an ERP method and attempted to explain why the framing effect is effective in the context of green marketing. In our ERP study, we found some components that were associated with message framing in green-marketing appeals. These components where found across three stages: in the first stage, the P1; in the second stage, the N170; and in the third stage, the P3. The findings for these three components are consistent with the hypothesis that the processing of the emotional content of message stimuli may occur in conjunction with attentional involvement. This was because these messages contain slogans and appeal to consumers to indulge in an environmentally safe action or to avoid performing an unsafe action.

Based on underlying neural mechanisms and brain processing, our results indicate that emotional information in green-marketing message framing can evoke ERP components in Pakistani consumers. The results suggest that subjects involve themselves in the messages, and that they clearly observe and understand the information given in the messages. Our participants focused on the threatening information as soon as it appeared. This suggests that, when they encounter environment-safety-related slogans and information, consumers apply both their attentional and emotional resources in their decision-making. Furthermore, in the context of green marketing, our findings suggest that consumers care more about environmental safety. The participants showed an interest in protective measures for the environment by showing greater inference from emotionally designed stimuli than neutral stimuli. Thus, it can be stated that, emotionally designed advertisements in the context of green marketing are more effective than are advertisements that feature neutral information for Pakistani markets.

There are some practical implications of this research. First, as both the positive and negative messages affected the participants, considering the nature of consumers, marketers should aim to construct their advertising claims based on positive and negative emotionally framed messages (Bresnahan et al., 2013; Zahid et al., 2018). Positively framed messages are likely to enhance consumers' engagement and motivate them to purchase environmentally safe products.

Second, negatively framed messages captured the initial attention of subjects, reflected by the early P1 amplitude. Using negative contents in advertising would help marketers secure the attention of consumers in the market at an early stage, which is likely to motivate the consumers to focus deeply on the advertised product.

Third, our research highlights the value and role of the neuroscience method in terms of studying consumers' immediate brain responses. Previous behavioral studies have examined message framing, but did not consider framing effects in the green marketing context. However, our results indicate that emotionally framed messages are effective in their role. Our findings clearly show that ERP is an effective method for studying consumers' motivational engagement with emotionally and non-emotionally significant marketing stimuli.

The current findings support the application of laboratory experiments to examine marketing and promotional activities. Neuromarketing-based Investigations of environmentally friendly advertising messages could improve researchers' understanding of the underlying neural mechanisms of the brain. Furthermore, these insights could help marketers by providing improved predictions of consumer behavior for targeted markets.

### 4.2 Limitations and directions for future research

The current study has some limitations that can be addressed in future studies. The message frames used in this study comprised a single factor: advertising a green product. However, different results may be obtained for advertising and consumer-related promotional activities that have more aspects, such as pricing and promotion at different marketing levels. The ERP results indicate that applying an emotionally-based framing effect is effective. Including specific emotionally attracting words that strongly encourage customers to consider helping the environment could be effective in specific market; such words may comprise feelings such as pride, guilt, and shame. Another limitation to this study was that all subjects were male students; in the future, studies examining the message-framing effect should perform their investigations using both male and female subjects, and with a broader age group. We used three types of simple messages in this study; further work examining the verbs, nouns, and other types of emotional information structure used in the green-marketing context could be conducted in future studies.

## Declarations

### Author contribution statement

M. Zubair: Conceived and designed the experiments; Performed the experiments; Analyzed and interpreted the data; Wrote the paper.

X. Wang: Conceived and designed the experiments; Analyzed and interpreted the data.

S. Iqbal and M. Awais: Analyzed and interpreted the data; Wrote the paper.

R. Wang: Performed the experiments; Analyzed and interpreted the data.

### Funding statement

This research did not receive any specific grant from funding agencies in the public, commercial, or not-for-profit sectors.

### Competing interest statement

The authors declare no conflict of interest.

### Additional information

No additional information is available for this paper.
